# Tolerance and response of afatinib combined with tegafur in patients with advanced biliary tract cancer after failure of gemcitabine-based systemic therapy

**DOI:** 10.1007/s12672-026-05039-4

**Published:** 2026-04-19

**Authors:** Zhizheng Li, Ping Li, Shaosheng Wu, Mei Zhang, Fengli Zhang, Lei Zheng, Li Su

**Affiliations:** 1 Anhui University of Traditional Chinese Medicine, Hefei, 230012 Anhui China; 2 Department of Integrated Traditional and Western Medicine in Oncology, The First Affiliated Hospital of Anhui Medical University, Hefei, 230000 Anhui China; 3 Center of Integrated Traditional and Western Medicine in Oncology, Anhui Medical University, Hefei, 230000 Anhui China

**Keywords:** Biliary tract cancer, Afatinib, Gemcitabine, Tegafur, Second-line treatment

## Abstract

**Background:**

Off-label afatinib with tegafur was attempted as a last resort option in a patient with advanced gallbladder cancer, achieving satisfactory outcomes. This study evaluated the benefits and toxicity of afatinib with tegafur in advanced biliary tract cancer (BTC).

**Methods:**

This retrospective study included patients who received afatinib combined with tegafur after failure of gemcitabine-based systemic therapy between Oct 2017 and Dec 2024 at the First Affiliated Hospital of Anhui Medical University. Progression-free survival (PFS), overall survival (OS), disease control rate (DCR), objective response rate (ORR), and treatment-related adverse events were evaluated. Cox regression analysis and subgroup analysis were used to assess the relationship between different factors and OS.

**Results:**

Fifty-eight patients were included. Two patients achieved partial response, 28 achieved stable disease, and 28 achieved progressive disease, indicating a DCR of 51.7% and an ORR of 3.4%. Median PFS and OS were 4.0 and 6.6 months, respectively. The toxicity associated with afatinib and tegafur was generally acceptable. OS was prolonged in six patients with positive HER-2 and four patients with positive KRAS (G13D) mutations (median: 11.0 months) compared with those without mutations (median: 6.30 months) or unknown genetic status (median: 6.30 months) (*P =* 0.013). The multivariable Cox analysis showed that *n*o factors were independently associated with OS (all *P* > 0.05).

**Conclusions:**

Afatinib with tegafur had an acceptable safety profile for advanced BTC after gemcitabine-based systemic therapy failure. The outcome of patients with low performance status and HER-2 or KRAS (G13D) mutations may be better than patients without the mutation.

**Supplementary Information:**

The online version contains supplementary material available at 10.1007/s12672-026-05039-4.

## Introduction

Biliary tract cancer (BTC) includes gallbladder cancer, intrahepatic BTC, extrahepatic BTC, and ampullary cancer [1]. The global incidence rate of BTC is relatively low, accounting for approximately 3% of all malignant tumors of the digestive system [1–4]. However, in recent years, its incidence rate has shown a significant upward trend, with the increase being particularly notable in gallbladder cancer [3, 4]. BTC management is multidisciplinary, but its prognosis is generally poor because the patients often present with advanced disease [1, 2], yet research efforts are progressively leading toward more effective tailored management [5].

The ABC-02 trial concluded that gemcitabine (GEM) combined with cisplatin (DDP) is superior to GEM single-agent chemotherapy in patients with BTC [6–8]. Nevertheless, there is no standard second-line option for patients with advanced BTC who fail first-line gemcitabine-based combination chemotherapy [1, 2]. Some studies explored second-line therapies such as S-1, anti-HER-2, anti-PD-1, anti-MEK, and anti-angiogenesis strategies, but these treatments’ benefits are limited [9].

Increasing evidence suggests that the EGFR, HER-2, and HER-3 proto-oncogenes are mutated and activated at a significant rate in BTC [9, 10], underscoring the importance of the ErbB signaling pathway in the pathogenesis of BTC. Afatinib is the first available irreversible ErbB family blocker and can block EGFR, HER2, and ErbB4 by covalent, irreversible binding [10, 11]. Since ErbB3 does not have a kinase domain, it cannot be directly blocked by afatinib, but afatinib was found to prevent ligand-dependent phosphorylation of ErbB3 [12]. A study showed that among two new HER2-amplified BTC cell lines (SNU-2670 and SNU-2773; established from patients with gallbladder cancer), SNU-2773 cells are sensitive to afatinib compared with HER2-negative BTC cell lines, but the specific targets of afatinib for BTC remain unclear [13].

Tegafur (FT) is an oral fluorouracil-based compound preparation that plays a significant role in the multimodal treatment strategy of BTC [14, 15]. Its importance has been increasingly supported by evidence in the adjuvant and advanced palliative settings. The JCOG1202 (ASCOT) study demonstrated that in Asian patients with BTC who underwent radical resection, postoperative adjuvant single-agent S-1 treatment increased the 3-year overall survival rate compared to the control group (simple observation) (77.1% vs. 67.6%), and reduced the risk of death by 31% (adjusted HR = 0.69, 95% CI: 0.51–0.94, one-sided *P =* 0.0080) [16]. The III phase JCOG1113 (FUGA-BT) trial indicated that for the first-line treatment of advanced BTC, the median overall survival (OS) of the GS regimen (gemcitabine combined with tegafur-gimeracil-oteracil) reached 15.1 months, which was comparable to the 13.4 months of the standard GC regimen (gemcitabine combined with cisplatin), establishing the non-inferiority of the GS regimen [17]. It provided high-level evidence for this combined regimen to be one of the standard choices for the first-line treatment of advanced BTC.

Off-label afatinib combined with tegafur was attempted as a last resort option in a patient with advanced gallbladder cancer who failed multiple chemotherapy lines, and her progression-free survival (PFS) reached 183 days. Subsequently, some patients with advanced BTC after treatment failure volunteered to receive afatinib and tegafur. The present study aimed to conduct a retrospective evaluation of afatinib and tegafur’s benefits and toxicity in advanced BTC. The results could provide a basis for a clinical trial of afatinib and tegafur in BTC.

## Methods

### Study design and patients

This retrospective case-series study included patients treated between October 2017 and December 2024 at the First Affiliated Hospital of Anhui Medical University. This study was approved by the the Ethics Committee of the First Affiliated Hospital of Anhui Medical University. The need for individual consent was waived by the committee because of the retrospective nature of the study.

The inclusion criteria were (1) advanced BTC [1, 2], (2) progression after at least one line of systemic therapy in accordance with the guidelines of the National Comprehensive Cancer Network (NCCN) [2], (3) histologically confirmed BTC stage IV, (4) received afatinib after failure to first-line treatment, (5) at least one measurable lesion and at least one measurement following the standards of the Solid Tumor Response Evaluation Criterion (RESIST) 1.1, (6) completion of at least one cycle of afatinib combined with tegafur, and (7) available data about tumor response and adverse events. The exclusion criteria were (1) pregnant or lactating women, (2) cardio-cerebrovascular disease, pulmonary fibrotic disease, or severe herpes, (3) liver, kidney, or hematopoietic system failure, (4) mental illness, (5) another uncured primary carcinoma, (6) the patient was receiving afatinib or tegafur and the other drug was added during the course of treatment, or (7) switched to monotherapy during the course of treatment.

### Afatinib combined with tegafur treatment

Some patients received related target gene detection after discussion with their physician. All included patients received afatinib. According to the scoring standard of the Eastern Cooperative Oncology Group (ECOG) and age, different doses of afatinib were selected. For patients with an ECOG score of 3 or *≥* 70 years, the dose was 30 mg/day. For patients with an ECOG score of *≤* 2 or < 70 years, the dose was 40 mg/day. Treatment could be interrupted, reduced to 30 mg once daily, or permanently discontinued if there were severe adverse events (SAEs). The dosage of tegafur was 40 mg/day. The combination treatment of afatinib, tegafur, and other drugs depended on the physician’s choice and the general condition of the patient. One treatment cycle was 28 days. The “other agents” referred to here are the drugs used for the symptomatic treatment of adverse reactions after they have occurred. If drug-related diarrhea occurred, the modified Banxia Xiexin decoction (MBXD) was given (Banxia 10 g, Huanglian 10 g, Huangqin 10 g, Ganjiang 10 g, Dangshen 10 g, and Dazao 5 g boiled in water, and 200 mL was taken each time orally, once a day after breakfast and dinner) [18]. When diarrhea did not show significant improvement, loperamide was administered. If there was drug-related neutropenia or febrile neutropenia, granulocyte colony-stimulating factor (G-CSF) treatment was given, and consideration was given to suspend or reduce the dosage of tegafur. According to the severity of anemia or thrombocytopenia, corresponding supportive treatments (such as erythropoietin, blood transfusion, etc.) were provided. If there was a drug-related rash, the LG09 Laoguancao external washing prescription was used to deal with the rash by external application to the affected area for about 30 min, 3–5 times a day (Laoguancao 30 g, kushen 20 g, baixianpi 20 g, and zicao 20 g) [19], followed by hydrocortisone butyrate ointment 30 min after LG09. If there was abnormal liver function, bicyclol (25 mg three times a day) or diammonium glycyrrhizinate (0.15 g/three times a day) could be given to protect the liver [20].

### Assessments

PFS, OS, disease control rate (DCR), and objective response rate (ORR) were evaluated. PFS was defined as the time from the start of afatinib and tegafur to disease progression or death, whichever came first. OS was defined as the time from the start of afatinib and tegafur to the date of death of any cause or the last follow-up visit. Tumor responses were assessed by both radiologists and oncologists after the first treatment cycle. Assessments were performed every two cycles or when the lesions’ length and diameter increased by > 20% or new lesions appeared, indicating tumor progression. The tumor responses were assessed according to RECIST 1.1 based on computed tomography (CT), magnetic resonance imaging (MRI), and/or positron emission tomography (PET)-CT. Tumor responses included complete response (CR), partial response (PR), stable disease (SD), or disease progression (PD). DCR was defined as CR + PR + SD. ORR was defined as CR + PR. Drug-related adverse events were graded according to the National Cancer Institute’s Common Terminology Criteria for Adverse Events, version 4.03 (NCI CTCAE v4.03). CTCAE v4.03 was used consistently across the study period to avoid bias from changes in grading versions during the retrospective interval. For this retrospective analysis, two independent investigators systematically extracted and rechecked all adverse events from the original records according to the study case report form to ensure grading accuracy. Any discrepancies were resolved by arbitration with a senior investigator. HER2 status was assessed by immunohistochemistry (IHC), with positivity defined as IHC 3 + or as IHC 2 + with amplification confirmed by fluorescence in situ hybridization (FISH). KRAS mutations (including G13D) were detected using next-generation sequencing (NGS) or ARMS-PCR in tumor tissue or circulating tumor DNA (ctDNA) from blood samples.

### Follow-up

The follow-up was censored on Jun 26, 2025. The patients were followed until disease progression and death. All patients were followed routinely every 2 months. Abdominal CT, tumor biomarkers, blood routine, and biochemical tests were reexamined every time. All data were extracted from the patient charts.

### Statistical analysis

Statistical analysis was performed using SPSS 27.0 (IBM, Armonk, NY, USA). Continuous variables were presented as means ± standard deviations. Categorical variables were presented as n (%). The survival analyses were conducted using the Kaplan-Meier method and analyzed using the log-rank test. Univariate and multivariable Cox regression analysis and subgroup analysis were used to assess the relationship between different factors and OS. Variables with *P*-values < 0.20 in the univariate analyses were included in the multivariable analysis, except for the afatinib dose, which was entered in the model irrespective of the univariate *P*-value. The hazard ratios (HRs), 95% confidence intervals (CIs), and statistical significance of factors were calculated. Two-sided *P*-values < 0.05 were regarded as statistically significant.

## Results

### Baseline characteristics of patients

Seventy-seven patients with advanced BTC received afatinib combined with tegafur between October 2017 and Dec 2024. Nineteen patients were excluded because they had taken afatinib or tegafur for fewer than one cycle (28 days). Therefore, 58 patients with advanced BTC were included. The clinicopathological features at the onset of the treatment are shown in Table 1.

**Table 1:** Baseline demographic and disease characteristics

Characteristics	Afatinib monotherapy (*n* = 58)
Age (years), median (range)	64 (39–85)
< 60	25 (43.1%)
≥ 60	33 (56.9%)
Sex, n (%)	
Male Female	28 (48.3%) 30 (51.7%)
ECOG PS, n (%)	
0 1 2 3	4 (6.9%) 17 (29.3%) 24 (41.4%) 13 (22.4%)
Metastatic sites, n (%)	
Lung Liver Pancreas Adrenal Abdominal cavity Retroperitoneal Pelvic cavity Bone Other lymph nodes	10 (17.2%) 33 (56.9%) 2 (3.4%) 2 (3.4%) 20 (34.5%) 15 (25.9%) 3 (5.2%) 5 (8.6%) 8 (13.8%)
Pathological type, n (%)	
Gallbladder Extrahepatic bile duct Intrahepatic bile duct Ampulla of Vater	23 (39.7%) 34 (58.6%) 1 (1.7%) 0
Gene status, n (%)	
Positive HER-2 expression	6 (10.3%)
Positive for KRAS (G13D) mutation	4 (6.9%)
Positive expression of genes other than HER-2 or KRAS (G13D) mutation	14 (24.1%)
Unknown gene expression/mutation status	34 (58.6%)
Initial dose of afatinib, n (%)	
40 mg 30 mg	28 (48.3%) 30 (51.7%)
Underlying diseases, n (%)	15 (25.9%)
Cancer pain, n (%)	34 (58.6%)
Surgery, n (%)	24 (41.4%)

ECOG: Eastern Cooperative Oncology Group; PS: performance status

### Tumor response

**Fig. 1:**
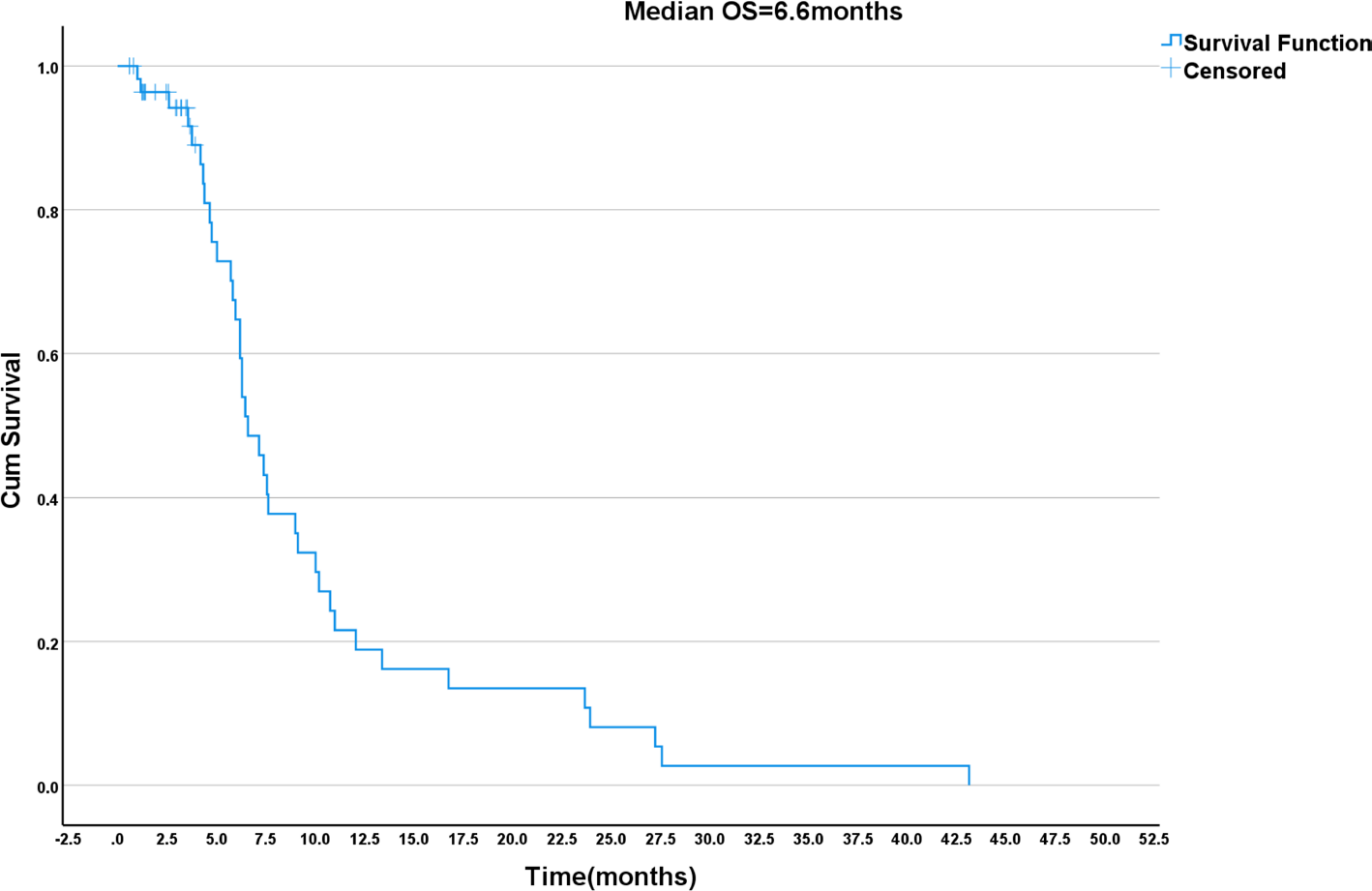
Kaplan-Meier estimates of progression-free survival (PFS) of patients with advanced biliary tract cancer treated with afatinib

**Fig. 2:**
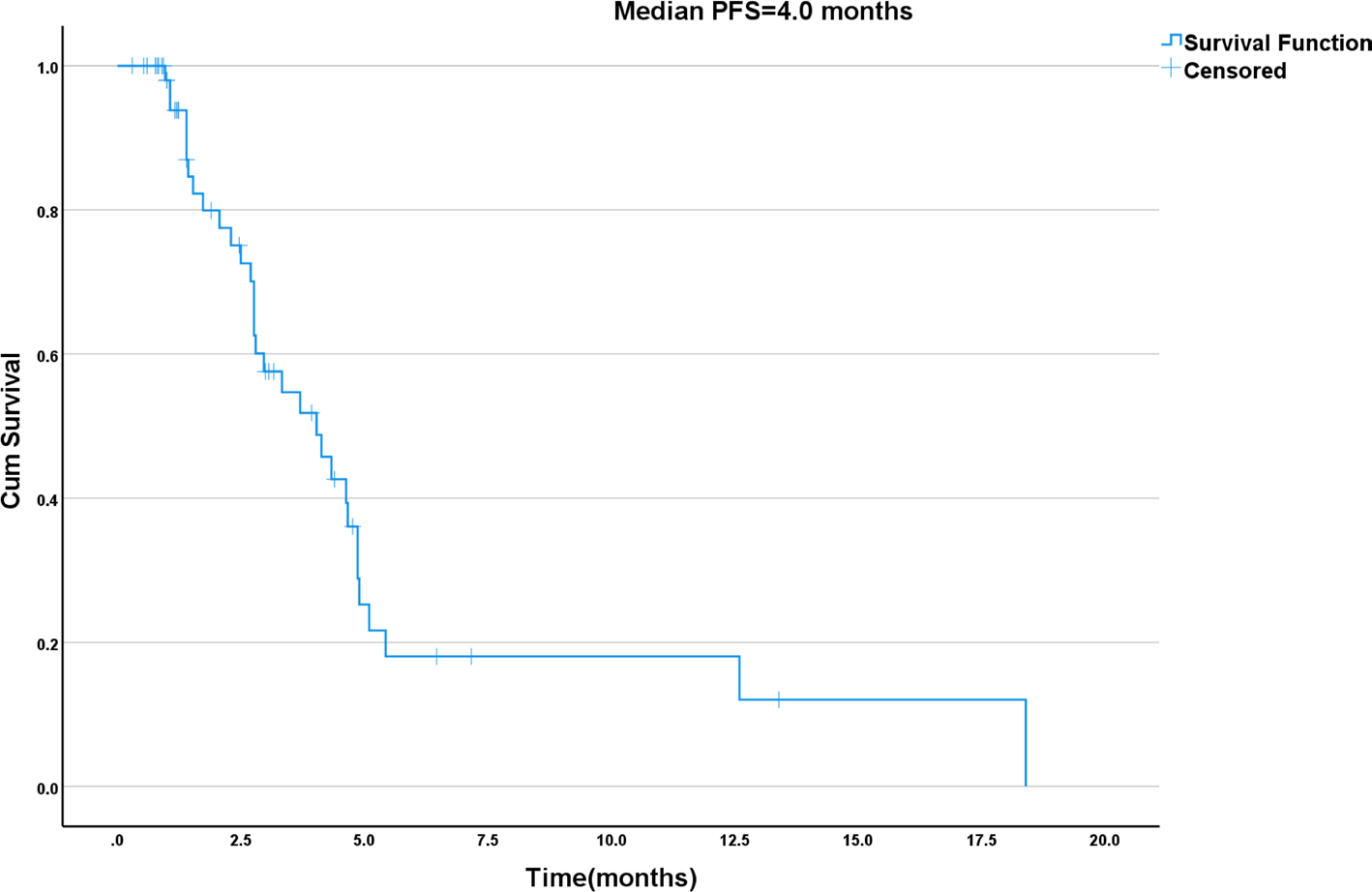
Kaplan-Meier estimates of overall survival (OS) of patients with advanced biliary tract cancer treated with afatinib

The median follow-up time for the entire cohort was 11.0 months (standard error: 2.7 months; 95% CI: 5.69–16.31 months). During follow-up, 30 patients were lost to follow-up; in the survival analyses, their data were censored at the date of last contact. Due to various factors and after discussion between the patients and their physicians, only 24 of the 58 patients had genetic testing: six patients were HER-2-positive, four patients had the KRAS mutation G13D, and 14 patients were negative. Again, after discussion, the patients who were negative for mutations decided to try afatinib combined with tegafur. At the end of follow-up, the median PFS and OS of the patients were 4.0 months (95% CI: 2.6–5.5) and 6.6 months (95% CI: 5.4–7.8), respectively. The Kaplan-Meier analyses of PFS and OS are shown in Figs. 1 and 2. The PFS was significantly longer in six patients with positive HER-2 expression and four patients with KRAS (G13D) mutation. Among 58 patients with an evaluable response, two patients (3.4%) achieved PR, 28 (48.3%) had SD, and 28 (48.3%) had PD, but no CR was achieved. Hence, the DCR was 51.7%, and the ORR was 3.4% (Supplementary Table S1).

As shown in Table 2, there were no significant associations of PFS or OS with age (*P =* 0.445 and *P =* 0.710, respectively), sex (*P =* 0.603 and *P =* 0.101, respectively), pathological type (*P =* 0.099 and *P =* 0.188, respectively), cancer pain (*P =* 0.178 and *P =* 0.723, respectively), comorbidities (*P =* 0.650 and *P =* 0.768, respectively), or dose (*P =* 0.234 and *P =* 0.343, respectively). Patients with ECOG performance status (PS) 0–1 had a better prognosis than those with ECOG PS 2–3 (median OS, 9.00 vs. 5.97, *P =* 0.003). Patients with positive HER-2 or KRAS (G13D) mutations had longer OS compared to those with no genetic alterations or unknown genetic status (median OS, 11.0, 6.30, 6.30 months, *P* = 0.013). The multivariable Cox analysis showed that *n*o factors were independently associated with OS, including sex (*P =* 0.316), ECOG PS (*P =* 0.060), afatinib dose (*P =* 0.929), or genetic status (*P* = 0.077 or 0.750) (Table 3 and Supplementary Figure S1). The results of the univariate and multivariable analyses should be considered exploratory pending confirmation in larger studies.

**Table 2:** Log-rank analysis of factors associated with PFS and OS in all patients with advanced BTC treated with afatinib

Variable	*n*	PFS (median, 95% CI)	OS (median, 95% CI)	*P*-value of PFS	*P*-value of OS
All patients	58	4.0 (2.6–5.5)	6.6 (5.4–7.8)		
Median age					
<60	25 (43%)	4.1 (2.3–5.9)	6.2 (5.5–6.9)	0.445	0.710
≥60	33 (57%)	3.7 (2.0-5.4)	7.4 (4.0-10.8)		
Sex					
Male	28 (48.3%)	3.0 (2.2–3.7)	7.6 (2.7–12.6)	0.603	0.101
Female	30 (51.7%)	4.6 (4.0-5.2)	6.3 (6.0-6.7)		
ECOG performance status					
0–1	20 (34.5%)	4.3 (2.3–6.4)	9.0 (5.7–12.3)	0.493	0.003
2–3	38 (65.5%)	4.0 (2.7–5.4)	6.0 (5.2–6.7)		
Pathological type					
Gallbladder	23 (39.7%)	3.3 (0.8–5.8)	13.0 (8.4–17.6)	0.099	0.188
Extrahepatic bile duct	34 (58.6%)	4.2 (3.2–5.2)	6.5 (5.2–7.8)		
Intrahepatic bile duct	1 (1.7%)	1.5 (0.0–0.0)	2.1 (0.0–0.0)		
Ampulla of Vater	0	0 (0.0–0.0)	0 (0.0–0.0)		
Gene status					
Positive of HER-2 or KRAS (G13D) expression	10 (17.2%)	4.3 (3.0-5.7)	11.0 (5.5–16.5)	< 0.001	0.013
No HER-2 or KRAS (G13D) mutation	14 (24.1%)	1.5 (0.5–2.6)	6.3 (5.1–7.5)		
Unknown gene expression/mutation status	34(58.6)	4.7(3.9–5.4)	6.3 (5.6-7.0)		
Underlying diseases					
Yes	43 (74.1%)	3.7 (2.1–5.3)	6.2 (4.9–7.6)	0.650	0.334
No	15 (25.9%)	4.1 (1.5–6.8)	6.6 (5.0-8.2)		
Cancer pain					
Yes	34 (58.6%)	2.8 (1.9–3.8)	6.3 (5.9–6.7)	0.178	0.723
No	24 (41.4%)	4.1 (3.4–4.9)	7.6 (6.1–9.1)		
Initial afatinib dose					
40 mg	28 (48.7%)	3.3 (2.2–4.5)	6.3 (5.7–6.9)	0.234	0.343
30 mg	30 (51.7%)	4.9 (3.8–5.9)	7.4 (3.4–11.4)		

**Table 3:** Univariate and multivariable Cox proportional hazards model for OS

	Univariate			Multivariable		
	HR	95% CI	*P*	HR	95% CI	*P*
Sex (male vs. female)	0.559	0.276–1.132	0.106	0.660	0.293–1.488	0.316
Age (< 60 vs. ≥60)	1.404	0.734–2.688	0.305	-	-	-
ECOG-PS (0 + 1 vs. 2 + 3)	0.296	0.127–0.690	0.005	0.402	0.156–1.040	0.060
Pain (no vs. yes)	0.886	0.453–1.733	0.724	-	-	-
Afatinib dose (40 vs. 30 mg)	0.723	0.369–1.418	0.346	1.034	0.492–2.175	0.929
Underlying diseases (no vs. yes)	0.685	0.315–1.487	0.338	-	-	-
Surgery (yes vs. no)	0.970	0.503–1.871	0.927	-	-	-
Lesion site (gallbladder cancer vs. cholangiocarcinoma)	1.243	0.615–2.513	0.544	-	-	-
Metastatic lesions (< 2 vs. *≥*2)	0.882	0.446–1.744	0.718	-	-	-
Genetic status (negative vs. positive)	0.199	0.058–0.677	0.010	0.279	0.077–1.141	0.077
Genetic status (not detected vs. positive)	0.714	0.294–1.737	0.458	0.843	0.295–2.411	0.750

Variables with *P*-values < 0.20 in the univariate analyses were included in the multivariable analysis, except for the afatinib dose, which was entered in the model irrespective of the univariate *P*-value

HR: hazard ratio; CI: confidence interval; ECOG-PS: Eastern Cooperative Oncology Group Performance Status

### Adverse events

Fourteen patients (24.1%) had dose modifications due to adverse events (Table 4). Differences in treatment exposure reveal the impact of toxicity on treatment patterns. Patients in the dose-adjustment group had a median duration of combination therapy of 6.2 months and a median number of cycles of 4.1, both substantially shorter than the standard-dose group (11.0 months and 5.3 cycles). At the same time, the incidence of grade *≥* 3 adverse events was higher in the dose-adjustment group (14.3% vs. 2.3% in the standard-dose group).

**Table 4:** Baseline Characteristics, Treatment Exposure and Outcomes by Subgroups Based on Treatment Dose Intensity

Project	All (*n* = 58)	Standard-dose group (*n* = 44)	Dose adjustment group (*n* = 14)
Baseline characteristics			
Age (years), median (range)	64 (39–85)	61.5 (39–80)	70 (54–85)
ECOG PS 2–3, n (%)	38 (65.5%)	27 (61.4)	11 (78.6)
Metastatic lesion ≥ 2, n (%)	31 (53.4%)	21 (47.7)	10 (71.4)
Exposure therapy			
Median combined treatment duration (months)	6.5 (5.3–7.6)	11 (1.21–20.79)	6.2 (0.00-13.32)
Median number of combined treatment cycles (range)	5.05 (1.07–46.21)	5.25 (1.64–46.21)	4.11 (1.07–25.93)
Therapeutic outcome			
Median progression-free survival (months)	4.0 (2.6–5.5)	4.1 (3.15–5.11)	3.3 (2.54–4.13)
Median OS (months)	6.6 (5.4–7.8)	7.4 (5.88–8.92)	6.2 (5.71–6.69)
Safety (grade ≥ 3 adverse events)			
Any grade ≥ 3 adverse events, n (%)	3 (5.2%)	1 (2.3%)	2 (14.3%)
Thyroiditis grade ≥ 3, n (%)	2 (3.4%)	0	2 (14.3%)
Diarrhea grade ≥ 3, n (%)	0	0	0
Neutropenia grade ≥ 3, n (%)	1 (1.7%)	1 (2.3%)	0

The adverse events associated with afatinib and tegafur were generally acceptable. Neutropenia, diarrhea, rash, stomatitis, pruritus, dry skin, epistaxis, and conjunctivitis were observed in 31.0%, 41.4%, 48.3%, 32.8%, 44.8%, 75.9%, 10.3%, and 15.5% of the cases, respectively (Table 5). Only two patients developed grade 3 thyroiditis, and one patient experienced grade 3 neutropenia. After reducing the dose of afatinib and administering G-CSF treatment, the symptoms were relieved. Grade 4 and 5 adverse events were not observed. Seventeen patients developed mild liver dysfunction, and they were treated with oral bicyclol (25 mg, three times a day).

**Table 5:** Treatment-related AEs by NCI-CTCAE grade

Adverse event	*n* (%)			
	Grade 1	Grade 2	Grade 3	All
Diarrhea	18 (31.0)	6 (10.3)	0	24 (41.4)
Stomatitis	15 (25.9)	4 (6.9)	0	19 (32.8)
Cheilitis	15 (25.9)	2 (3.4)	0	17 (29.3)
Peptic bleeding	2 (3.4)	2 (3.4)	0	4 (6.9)
Rash/acneiform dermatitis	22 (40.0)	6 (10.3)	0	28 (48.3)
Pruritus	22 (37.9)	4 (6.9)	0	26 (44.8)
Dry skin	38 (65.5)	6 (10.3)	0	44 (75.9)
Paronychia	6 (10.3)	2 (3.4)	2 (3.4)	10 (17.2)
Cystitis	4 (6.9)	0	0	4 (6.9)
Epistaxis	6 (10.3)	0	0	6 (10.3)
Rhinorrhea	4 (6.9)	0	0	4 (6.9)
Pyrexia	2 (3.4)	0	0	2 (3.4)
Conjunctivitis	7 (12.1)	2 (3.4)	0	9 (15.5)
Neutropenia	12 (20.7)	5 (8.6)	1 (1.7)	18 (31.0)
Red blood cell reduction	6 (10.3)	0	0	6 (10.3)
Thrombocytopenia	4 (6.9)	2 (3.4)	0	6 (10.3)
Direct bilirubin increased	3 (5.2)	1 (1.7)	0	4 (6.9)
Indirect bilirubin increased	2 (3.4)	1 (1.7)	0	4 (6.9)
ALT increased	6 (10.3)	2 (3.4)	0	8 (13.8)
AST increased	7 (12.1)	2 (3.4)	0	9 (15.5)

AE: adverse event; ALT: alanine aminotransferase; AST: aspartate aminotransferase; NCI-CTCAE: National Cancer Institute Common Terminology Criteria for Adverse Events

^a^There were no Grade 5 treatment-related AEs

With respect to Western supportive care, six patients (10.3%) received loperamide for diarrhea, and six patients (10.3%) were treated with granulocyte colony-stimulating factor (G-CSF) for neutropenia. Regarding TCM-based interventions, 24 patients (41.4%) received oral modified Banxia Xiexin decoction for diarrhea, 28 patients (48.3%) were treated for skin rash with topical Geranium wilfordii wash combined with hydrocortisone butyrate ointment, and 17 patients (29.3%) received oral bicyclol for abnormal liver function.

## Discussion

Gemcitabine-based chemotherapy is the treatment of choice for BTC, but there is no standard second- or later-line therapy. This study evaluated the benefits and toxicity of afatinib combined with tegafur in advanced BTC. The results suggest that afatinib combined with tegafur might be a reasonable option with an acceptable safety profile for advanced BTC after failure of gemcitabine-based systemic therapy. Given the high loss-to-follow-up rate, retrospective nature of the study, and small sample size, these findings should be confirmed in future research.

For patients with advanced BTC, a gemcitabine-based chemotherapy regimen is recommended by the NCCN guidelines as the standard first-line treatment [2]. The options for second-line treatment are limited. Indeed, the median OS of the mFOLFOX regimen is only 6.2 months, and the efficacy is poor [21, 22]. The NIFTY study suggested that liposomal irinotecan combined with 5-fluorouracil (NALIRIFOX) can be an alternative option [23]. Other new drugs targeting gene alterations such as FGFR, IDH1, HER2, and BRAF have shown activity in the second- or later-line treatment of advanced BTC, but are only applicable to a small number of patients selected through molecular screening [24–26]. The literature highlights the need for a new targeted drug or regimens of combination therapies as a second-line treatment option.

Afatinib is an oral, irreversible ErbB family blocker that inhibits signaling from all ErbB receptor homodimers and heterodimers [27]. Tegafur (S-1) is a fluorouracil precursor drug. By continuously inhibiting thymidylate synthase (TS) to interfere with DNA synthesis, it holds a significant position in the treatment of BTC [28]. The present study showed two patients with a PR, 28 with an SD, and 28 with a PD, according to RECIST 1.1. Nevertheless, the PFS was significantly prolonged in six patients with BTC with positive HER-2 expression and four patients with positive KRAS mutation; the association was observed in the Kaplan-Meier analyses but not in the multivariable analysis. Afatinib has a certain effect on lung cancer, breast cancer, urothelial carcinoma, and colorectal cancer with positive HER-2 expression [29, 30]. Therefore, the detection of actionable genes in patients with BTC is also important. Efforts to determine sensitive mutations are needed for selecting targeted therapies and maximizing (although not guaranteeing) the chances of efficacy. The present study also showed that ECOG PS 2–3 was associated with OS in the Kaplan-Meier/log-rank and univariate Cox analyses, as supported by previous studies [31, 32], but the association was not independent after adjustment for other factors in the multivariable analysis. However, there is currently no published clinical evidence for afatinib combined specifically with tegafur in BTC management; the available literature only supports their use individually or in other combinations [33–36]. Additional studies are necessary to examine the benefits of the combination in BTC.

Afatinib plus tegafur showed disease control and survival outcomes that were broadly comparable to published second‑line fluoropyrimidine‑based regimens for advanced BTC after gemcitabine-platinum, with a potential signal of improved benefit in HER2‑ or KRAS(G13D)‑positive disease. These data suggest that this regimen may be a reasonable second‑line option, particularly for molecularly selected patients, while acknowledging the absence of randomized comparisons. In the present study, treatment with afatinib plus tegafur yielded a DCR of 51.7% and an ORR of 3.4%, with median PFS and OS of 4.0 and 6.6 months, respectively. In large retrospective series of second‑line fluoropyrimidine‑based chemotherapy after gemcitabine-cisplatin failure, median PFS and OS have generally ranged from approximately 1.9–3.2 months and 6.5–7.7 months, with low response rates (typically 5%-15%) despite combination regimens such as FOLFOX, FOLFIRI, or fluoropyrimidine-platinum [37–40]. Thus, the disease control and survival outcomes observed with afatinib plus tegafur appear broadly comparable to those reported for standard second‑line chemotherapy in unselected BTC populations, although cross‑trial comparisons must be interpreted cautiously. Of note, patients with HER2‑positive or KRAS G13D‑mutant tumors in our cohort experienced numerically longer survival (median PFS of 4.3 months and OS of 11.0 months) than those without these alterations (median PFS of 1.5 months and OS of 6.3 months), suggesting that dual ErbB blockade with afatinib in combination with fluoropyrimidine may be particularly active in biomarker‑defined subgroups. This observation is consistent with emerging data supporting HER2‑directed strategies in advanced BTC, in which dual HER2 blockade (pertuzumab plus trastuzumab) achieved an ORR of 23%, median PFS of 4.0 months, and median OS of 10.9 months in HER2‑positive disease [37, 40–42]. Collectively, these findings support further prospective evaluation of afatinib‑based combinations as a second‑line option, possibly in HER2‑ or KRAS‑driven BTC, while recognizing that randomized trials against contemporary standards are needed to definitively establish their relative efficacy.​.

Furthermore, fluoropyrimidine-platinum combinations such as FOLFOX have become a reference backbone in gastrointestinal malignancies, including biliary tract cancer and metastatic gastric/GEJ adenocarcinoma, where modified FOLFOX‑6 has demonstrated efficacy comparable to docetaxel-cisplatin-5‑fluorouracil (mDCF) but with a more favorable toxicity profile [43–46]. In this context, the outcomes observed with afatinib plus tegafur (DCR 51.7%, median PFS 4.0 months, median OS 6.6 months) are within the range reported for fluoropyrimidine‑based doublets, while offering a targeted, biomarker‑oriented approach that yielded numerically longer survival in patients with HER2‑positive or KRAS G13D‑mutant tumors.

In most studies, the adverse reactions to afatinib and tegafur mainly include diarrhea, rash, and neutropenia, which may lead to a reduction in the dosage of afatinib or tegafur, or even suspension or early discontinuation of treatment [18, 47, 48]. Therefore, preventing and managing the adverse events caused by afatinib and tegafur are extremely important. TJ-14 showed a trend in reducing the risk of afatinib-induced diarrhea, and minocycline reduced the risk of afatinib-induced skin rash [49]. BXD is reported to prevent and control CPT-11-induced delayed diarrhea [18, 50]. In this study, the drug composition of the BXD mixture was adjusted by adding three Chinese herbal medicines (*Geranium wilfordii*, *Cortex dictamni*, and *Scrophularia*). *Geranium wilfordii* exhibits antioxidant, anti-inflammatory, and antitumor effects, which have a certain preventive effect on diarrhea and rash [51]. *Cortex dictamni* exhibits antibacterial, anti-inflammatory, anti-allergic, and antitumor, as well as other pharmacological activities, with the anti-inflammatory activity being particularly strong [52]. *Scrophularia* has anti-oxidation, anti-inflammatory, enzyme inhibition properties, and antitumor effects [53]. Neutropenia can be effectively controlled by granulocyte colony-stimulating factor (G-CSF) [54]. The rates of adverse reactions from afatinib and tegafur, including diarrhea (41.4%), rash (48.3%), and neutropenia (31.0%), were relatively low, and the patients generally had a good tolerance to this treatment. At the same time, MBXD may help afatinib achieve a better antitumor effect by reducing the need for dose adjustment or delay. Trials will be necessary to delineate the role of MBXD in the control of adverse events.

The apparently paradoxical finding of a higher incidence of grade *≥* 3 adverse events in the dose-adjustment group actually reflects the underlying logic: patients were assigned to the dose-adjustment group because they experienced intolerable toxicity (or had high-risk features) early in treatment, prompting clinicians to reduce the dose or interrupt therapy sooner. Thus, the shorter treatment duration and fewer cycles in this group are a direct consequence of poorer baseline tolerance or earlier onset of significant toxicity, and toxicity drove the reduction in exposure. The patient characteristics support this explanation. The patients in the dose-adjustment group were older (median 70 years vs. 61.5 years), had worse performance status (ECOG PS 2–3: 78.6% vs. 61.4%), and carried a higher metastatic burden (*≥* 2 metastatic sites: 71.4% vs. 47.7%). These factors are typically associated with a lower threshold for treatment-related toxicity. Therefore, even after dose adjustment, this group remained at a higher risk of severe adverse events. In the present study, the standard-dose group received longer treatment but showed a very low rate of severe toxicity. It suggests that for patients who can tolerate the initial standard dose, extending treatment duration did not result in a meaningful accumulation of severe toxicities. The toxicity profile of the combination regimen appears to manifest predominantly in the early treatment period (for example, during the initial cycles); patients who successfully pass through this early phase and continue therapy tend to have favorable tolerability.

In the present study the multivariable Cox model included four variables (five degrees of freedom) and therefore lies at the lower end of commonly recommended sample-size guidance for Cox regression. Indeed, conventional guidance suggests at least 10 outcome events per variable (EPV) to reduce overfitting, bias, and instability of regression coefficients, though more recent work shows that acceptable performance can sometimes be achieved with lower EPV, depending on the setting [55, 56]. At the same time, simulation studies indicate that when low-prevalence predictors are included (e.g., a small HER2/KRAS-positive subgroup), higher EPV (*≥* 20) may be needed to avoid bias and imprecision. Therefore, considering that the multivariable model contains four variables (five degrees of freedom), the model would be appropriate but on the edge of the limit for reliability. Nevertheless, the results of the univariate and multivariable analyses should be considered exploratory.

A strength of the study could be the detailed description of adverse event management, including supportive measures, which adds practical value for clinicians and should be highlighted as a strength of the study. Still, the use of TCM for patient support could be regarded as a limitation in Western settings.

The limitations of this study were its retrospective design, the small sample size, potential missing data, possible information bias, and the lack of a control group. The loss-to-follow-up rate was high (30 patients). Although follow-up was censored on the date of the last visit, it limits the data available for those patients. The quality of life could not be fully assessed based on retrospective data. There was heterogeneity in the timing and reasons for dose adjustments. The detection of gene mutations may better explain the effect of afatinib on advanced BTC patients. However, only 24 patients received gene detection in this study. This retrospective study only shows real-world data that need to be validated using double-blind clinical trials and further follow-up. The hypothesis-generating character of the findings must be highlighted, and prospective biomarker-driven trials are necessary to improve BTC management. In addition, future prospective studies should record the timing of the first occurrence of grade *≥* 2 toxicity and the specific triggers for dose modification in greater detail and should develop predictive models to identify high-risk patients who may benefit from preemptive dose reduction or intensified supportive care. Drug-related adverse events were graded according to NCI CTCAE v4.03. Although CTCAE v6 is now available, CTCAE v4.03 was the version being used during the study period. It was kept to remain consistent across the study period and to avoid interpretation bias from changes in grading versions based only on retrospective data. Of note, the core toxicities (e.g., diarrhea and rash) are consistent among CTCAE versions 4, 5, and 6 (https://dctd.cancer.gov/research/ctep-trials/trial-development/ctcae-v6.0.xlsx for comparison of versions 5 and 6). Such efforts would enable individualized exposure management to better balance efficacy and safety.

In conclusion, afatinib combined with tegafur shows encouraging benefits and tolerability and might be an option with an acceptable safety profile for patients with BTC after failure of first-line gemcitabine treatment. This treatment may be more effective in patients with positive HER-2 and KRAS (G13D) genetic status, but confirmation is required. These results lay the foundation for future studies and trials, but the results need to be validated, considering the high loss-to-follow-up rate, retrospective nature of the study, and small sample size.

## Supplementary Information

Below is the link to the electronic supplementary material.

Supplementary Material 1.

## Data Availability

The data could not be openly available due to local policy, and could only be available from the corresponding author upon reasonable request.
