# Supplementary material for: Tolerance and response of afatinib combined with tegafur in patients with advanced biliary tract cancer after failure of gemcitabine-based systemic therapy
Source: Discov Oncol. 2026 Apr 19;17:824. doi: 10.1007/s12672-026-05039-4 (PMC13222911; doi:10.1007/s12672-026-05039-4)
Supplement: Supplementary file 1 — Supplementary Material 1. [file 12672_2026_5039_MOESM1_ESM.docx]

**Supplementary materials**

**Table S1.** Tumor response of patients with advanced BTC treated with afatinib combined with tegafur-oxidized glucose.

| Response (n=58) | n (%) |
| --- | --- |
| CR | 0 |
| PR | 2 (3.4%) |
| SD | 28 (48.3%) |
| PD | 28 (48.3%) |
| ORR | 2 (3.4%) |
| DCR | 30 (51.7%) |

CR: complete response; PR: partial response; SD: stable disease; PD: progression disease; ORR: objective response rate (CR + PR); DCR: disease control rate (CR + PR + SD).


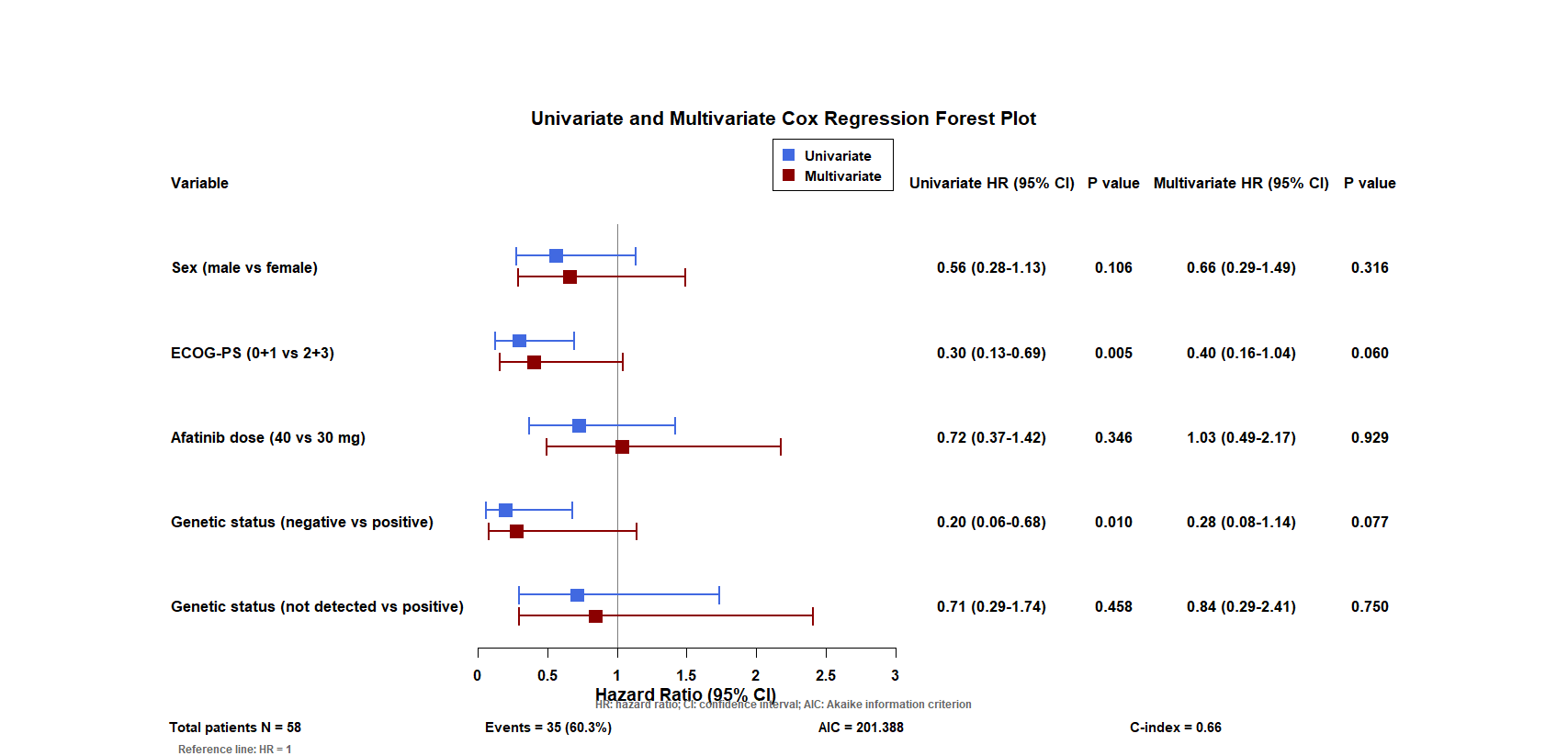


**Figure S1.** Subgroup analysis of overall survival rates of patients with advanced biliary tract cancer who received afatinib and tegafur-uracil treatment.
